# Above-ground biomass references for urban trees from terrestrial laser scanning data

**DOI:** 10.1093/aob/mcab002

**Published:** 2021-03-10

**Authors:** Daniel Kükenbrink, Oliver Gardi, Felix Morsdorf, Esther Thürig, Andreas Schellenberger, Lukas Mathys

**Affiliations:** 1 Swiss Federal Institute WSL, Zürichstrasse 111, CH-8903 Birmensdorf, Switzerland; 2 Remote Sensing Laboratories, University of Zurich, Winterthurerstrasse 190, CH-8045 Zurich, Switzerland; 3 School of Agricultural, Forest and Food Sciences HAFL, Länggasse 85, CH-3052 Zollikofen, Switzerland; 4 Federal Office for the Environment FOEN, CH-3003 Bern, Switzerland; 5 Nategra LLC, Nydeggstalden 30, CH-3011 Bern, Switzerland

**Keywords:** TLS, non-forest trees, urban trees, AGB, wood volume, biomass, allometry, tree structure, QSM, point cloud

## Abstract

**Background and Aims:**

Within extending urban areas, trees serve a multitude of functions (e.g. carbon storage, suppression of air pollution, mitigation of the ‘heat island’ effect, oxygen, shade and recreation). Many of these services are positively correlated with tree size and structure. The quantification of above-ground biomass (AGB) is of especial importance to assess its carbon storage potential. However, quantification of AGB is difficult and the allometries applied are often based on forest trees, which are subject to very different growing conditions, competition and form. In this article we highlight the potential of terrestrial laser scanning (TLS) techniques to extract highly detailed information on urban tree structure and AGB.

**Methods:**

Fifty-five urban trees distributed over seven cities in Switzerland were measured using TLS and traditional forest inventory techniques before they were felled and weighed. Tree structure, volume and AGB from the TLS point clouds were extracted using quantitative structure modelling. TLS-derived AGB estimates were compared with AGB estimates based on forest tree allometries dependent on diameter at breast height only. The correlations of various tree metrics as AGB predictors were assessed.

**Key Results:**

Estimates of AGB derived by TLS showed good performance when compared with destructively harvested references, with an *R*^2^ of 0.954 (RMSE = 556 kg) compared with 0.837 (RMSE = 1159 kg) for allometrically derived AGB estimates. A correlation analysis showed that different TLS-derived wood volume estimates as well as trunk diameters and tree crown metrics show high correlation in describing total wood AGB, outperforming tree height.

**Conclusions:**

Wood volume estimates based on TLS show high potential to estimate tree AGB independent of tree species, size and form. This allows us to retrieve highly accurate non-destructive AGB estimates that could be used to establish new allometric equations without the need for extensive destructive harvesting.

## INTRODUCTION

Urban areas are predicted to increase in size as a fraction of total land cover (Seto *et al.*, 2012; Hutyra *et al.*, 2014). With this increase, alongside ongoing climate change, the role of urban trees becomes increasingly important, providing well-recognized ecosystem services. These include mitigation of the urban ‘heat island’ effect and local climate regulation (Armson *et al.*, 2012), their importance in biodiversity conservation (Goddard *et al.*, 2010) and abating air pollution (Baró *et al.*, 2014), and their benefits for well-being and aesthetics (Kardan *et al.*, 2015). The importance of forests as a major carbon sink is well established, and methods of measuring and assessing the capability of forests to sequester atmospheric CO_2_ are abundant. The role of non-forest trees and urban forests in the global carbon cycle is still largely unknown and understudied. Urban trees show large potential to sequester significant amounts of carbon from the atmosphere; this carbon is absorbed into plant tissue through photosynthesis, building an important carbon sink. Preliminary estimates for the Swiss greenhouse gas inventory based on the work of Price *et al.* (2017) show that 9.4 million tonnes of carbon are stored in trees within settlements in Switzerland. However, due to the relatively small spatial area of urban trees in terms of global forest cover, their contribution to in the global carbon cycle is given little consideration (Churkina, 2016). It is therefore important to develop and analyse methods to monitor and assess urban trees and the valuable ecosystem functions they provide, including, but not limited to, carbon storage. As urban trees encounter environmental and neighbourhood circumstances different from those of their forest counterparts, they often show a higher dynamic and plasticity with higher mortality (Tigges *et al.*, 2017) and often faster growth rates (Pretzsch *et al.*, 2017), and they tend to develop relatively large crowns and branches (MacFarlane and Kane, 2017). Therefore, methods to assess tree structure and their ecosystem services developed for natural ecosystems may not be transferable to urban areas (McHale *et al.*, 2009). Consequently, extensive studies on ecosystem services provided by urban trees, such as carbon storage capabilities, are highly necessary.

A reliable way to assess carbon storage and other ecosystem functions of trees is the measurement of above-ground biomass (AGB), which is defined as ‘the above ground standing dry mass of live or dead matter from tree or shrub (woody) life forms, expressed as a mass per unit area’ (CEOS, 2020). With trees accounting for up to 97 % of urban AGB (Davies *et al.*, 2011), it is of major interest to get accurate estimates of the AGB stored in them. However, AGB can only be directly measured with destructive harvesting, which is an expensive and time-consuming approach often not applicable to larger numbers of trees. Therefore, AGB is often estimated with the use of allometric equations, relying on non-destructive and more easily measurable tree metrics, such as tree height, diameter at breast height (DBH, measured 1.3 m above the ground), crown height or projected crown area. However, the establishment of accurate species- and ecosystem-specific allometric equations requires extensive destructive sampling (Roxburgh *et al.*, 2015), which is often not practical or too expensive in urban environments. On the other hand, allometric equations developed for forest trees may often not be transferable to urban trees (McHale *et al.*, 2009).

Light detection and ranging (LiDAR), particularly terrestrial laser scanning (TLS), has received great attention for assessing 3-D tree structure and AGB in both forests and urban areas (Disney *et al.*, 2018). Due to the increased fidelity of recent developments in TLS instruments, increasing detail of the complex tree structure and physically based semantics of the tree can be extracted (Disney *et al.*, 2018; Morsdorf *et al.*, 2018). With an approach known as quantitative structure modelling (QSM) as developed by Lefsky and McHale (2008) and Raumonen *et al.* (2013), in which cylinders are fitted to the 3-D point cloud, detailed information on tree volume and structure can be extracted independent of tree size and shape (Disney *et al.*, 2018). Calders *et al.* (2015) and Gonzalez de Tanago *et al.* (2018) have shown that these methods are able to estimate tree AGB to within 10 % of the value measured by destructive harvesting of the tree. When species-specific allometries were applied, up to >35 % underestimation was found for large tropical trees (Gonzalez de Tanago *et al.*, 2018) and eucalypt trees (Calders *et al.*, 2015). With the highly accurate estimation of wood volume and AGB provided by these methods, new allometric models could be established without the need for destructive sampling of trees, as was shown by Wilkes *et al.* (2018). However, the quality of such volumetric tree models is highly dependent on the complexity of the trees and the surrounding area, scan acquisition geometry, pattern and settings (Abegg *et al*., 2017; Schneider *et al.*, 2019), and meteorological conditions during the LiDAR scans. So far, data on the amount and distribution of such detailed tree models from which new allometric models could be derived are still relatively scarce. In this study 55 urban trees distributed over eight cities in the Swiss plateau were measured using traditional forest inventory techniques (DBH, tree height, crown dimensions etc.) and with a terrestrial laser scanner. The high-resolution 3-D point cloud was analysed to extract detailed information about tree structure, wood volume and AGB. The trees were professionally felled and weighed to validate estimated tree AGBs, both for those based on allometric equations and for those based on TLS data. In this study we evaluate and validate TLS and traditional tree inventory techniques to assess tree structure and AGB of urban trees. We further highlight TLS as a tool for non-destructive AGB retrieval, which could potentially help in building new allometric models, specifically designed for AGB estimation of urban trees.

## MATERIALS AND METHODS

### Study area

Twenty-seven percent of Switzerland is covered by trees, with 6.1 % of the trees located outside forest boundaries (Ginzler *et al.*, 2011). Urban areas are the predominant locations of non-forest trees in Switzerland. In order to get representative coverage of the urban tree population in Switzerland, multiple cities were asked prior to the tree felling season during the winter months to provide information about the trees to be felled. The selection criteria were based on tree species, size and age. The aim was to select trees to represent the Swiss non-forest tree population based on these three criteria as best as possible given the operational constraints. A total of 55 trees were measured and felled, distributed over seven cities (Bern 15, Biel 9, Lausanne 15, Lucerne 6, Winterthur 2, Solothurn 5, Zurich 3). A complete list of the trees is given in Appendix Table A1.

### TLS data acquisition

High-resolution TLS point-cloud data for each tree were acquired using a Riegl VZ-1000 scanner (Riegl, Austria) operated at a wavelength of 1550 nm, a pulse repetition frequency of 150 kHz with a maximum range of 950 m, and a pulse sampling interval of 0.02°. The scanner has a beam divergence of 0.3 mrad, resulting in an increase of 30 mm in beam diameter per 100 m distance. A field of view of 100° in the vertical direction and 90–180° in the horizontal direction was used, adapted to the plot characteristics and number of scans per plot. For each tree, three or four scan positions ~20–30 m away from the tree position were chosen depending on the complexity and size of the tree. Reflecting cylindrical targets were distributed within the plot area to aid the co-registration of scans from different positions. The combination of multiple scans reduces occlusion effects (Abegg *et al*., 2017; Kükenbrink *et al.*, 2017), and to some extent homogenizes the point density of the resulting point-cloud data. The laser acquisitions for each plot took between 1 and 2 h, including setup and breakdown, and produced unfiltered point clouds of up to 45 million points per scan position.

### Reference data acquisition

For all 55 trees, DBH 1.3 m above ground was measured using a diameter tape and the tree height and crowning height were measured from three angles using a rangefinder (TruePulse 360). The crown width was measured on two orthogonal axes using an ordinary measuring tape. Once the tree was felled, the total weight of the tree was measured using a truck scale or a crane scale. The total weight was further divided into coarse wood (trunk and branches with diameter >7 cm) and fine wood (branches and twigs with diameter <7 cm). Multiple wood samples were taken from different compartments of each tree (tree trunk at breast height, trunk at half height, coarse branches >7 cm, branches 4–7 cm, small branches 1–4 cm and twigs <1 cm in diameter) in order to determine water content and basic wood density in the laboratory.

The wood water content was determined by weighing the fresh wood samples of the different tree compartments using a calibrated laboratory scale, then drying the samples for 96 h in an oven at 105 °C before their dry weight was measured using the same scale. The water content of all the samples (WC) was then derived with eqn (1):


$$\begin{array}{l} WC\;=\;(FW-DW)/FW \end{array}$$ (1)

where FW is fresh weight and DW is dry weight. The biomass (BM) of both the coarse wood and the fine wood of the reference trees was determined with eqn (2):


$$\begin{array}{l} BM=(1-WC)\times weight \end{array}$$ (2)

where weight is the fresh weight of the tree’s coarse wood or fine wood.

The basic wood density was determined by soaking the fresh samples in water for 24 h before their volume was determined using the water displacement method, i.e. by measuring the weight of the water that is displaced by complete submersion of the sample with a laboratory scale. The samples were then dried for 96 h at 105 °C to determine the dry weight. The basic wood density (WD) could then be determined with eqn (3):


$$\begin{array}{l} WD=DW/FV \end{array}$$ (3)

where DW is the dry weight and FV the fresh volume of the sample.

### Tree metric extraction from TLS point cloud

The raw point clouds from the different scan positions were co-registered based on the reflective cylindrical targets within the software RISCAN PRO version 2.0.2 r7440 (Riegl, Austria). Fine registration of the point clouds was performed using the multi-station adjustment tool provided in the RISCAN PRO software package. A final co-registration error (standard deviation of residuals) of the aligned point clouds of <1 cm was achieved for all plots. Laser returns with very low reflectance caused by atmospheric effects (e.g. fog or other small particles in the air) or by partial hits of the laser pulse (e.g. border of branches, leaves; see also Abegg *et al*., 2020a) were removed. The point cloud was further manually filtered to the extent that only laser returns from the main tree remained. As some trees measured in early winter still had some leaves on them, these leaves were manually filtered using the radiometric information on the laser returns, allowing for crude wood–leaf separation due to the different reflectance characteristics of the two materials. A wood–leaf separation based on geometric properties of the close neighbourhood of each laser return, as implemented, for example, in the LeWoS MATLAB tool (Wang *et al.*, 2020) or in the python library TLSeparation (Vicari *et al.*, 2019), was not performed, considering the few trees that still bore leaves.

Basic tree variables, such as the tree height, crown diameter, crown length (vertical extent from crown base to crown top), crown cover and crown volume, were extracted directly from the point cloud. Wood volume per compartment (diameter >7 cm and diameter ≤7 cm), DBH and trunk diameters at multiple heights above ground were extracted by fitting cylindrical objects into the point cloud following the QSM approach introduced by Raumonen *et al*. (2013, 2015), as implemented in the TreeQSM tool for MATLAB, version 2.3.1 (Raumonen *et al.*, 2013). For each tree the optimum parameter combination for the QSM model was found by running the optimum parameter selection approach as implemented in TreeQSM, in which multiple QSM runs with varying parameter sets (varying minimum and maximum patch sizes of cover sets) are performed (Raumonen *et al.*, 2013). TreeQSM finds the optimum parameter combination with the smallest divergence in the resulting QSM models between model runs. The optimal parameter combination was subsequently run 30 times in order to also obtain an estimate of the variability of the QSM model due to a random term within the QSM approach (Raumonen *et al.*, 2013). The resulting wood volumes for the two compartments were then multiplied by the basic wood density for the respective compartment as measured in the laboratory (see see Reference data acquisition in the Materials and Methods section) to obtain their AGB. Total tree AGB was then calculated from the sum of coarse and small wood AGB.

The tree metrics derived by the QSM or directly from the point cloud and from allometric equations were compared with the reference values measured *in situ* (see Reference data acquisition in the Materials and Methods section). We used linear regression to compare the different derived tree metrics with the reference measurements. We calculated the *R*^2^ value of the linear regression between reference and estimated values and their root mean square error (RMSE) with respect to the 1:1 line to evaluate the deviation from the reference data, following Calders *et al.* (2015). The bias was calculated to highlight whether the different methods under- or overestimate the reference values.

### Comparison with allometry-based AGB estimates

Estimates of AGB based on the QSM approach were compared with AGB estimates based on species- and region-specific allometric equations. For this purpose, species-specific allometric tariff models established for the Swiss National Forest Inventory (NFI) published in Kaufmann (2001) and Herold *et al.* (2019) were used to derive stem volumes. These tariff models were established for forest trees. The tariff volume over bark $\hat{Y}$ is estimated according to the following model:


$$\begin{array}{l} \hat{Y}=\exp\left(b_{0}+\;b_{1}\ln\left(d_{1.3}\right)+\;b_{2}\ln^{4}\left(d_{1.3}\right)+\;\underset{i=3}{\overset{7}{\sum }}\;b_{i}B_{i}\right) \end{array}$$ (4)

The index *i* corresponds to the additional single tree and sample-plot attributes (3 … 7), and *d*_1.3_ is the measured diameter at breast height. *B*3 to *B*7 are the following additional single tree and sample-plot attributes:

$B_{3}$ TMI: site quality expressed as the maximum of the total mean increment from stand establishment until the age of 50 years, in kilograms of dry weight per hectare per year. This was set to 4500 kg ha^−1^ year^−1^, corresponding to the average value found for forests in the Swiss plateau.

$B_{4}$ *d*_dom_: dominant diameter, i.e. mean diameter of the 100 thickest trees per hectare. As this is not applicable to the trees in our study, the DBH of the measured tree is used.

$B_{5}$ bifurcation of the stem (0 = no bifurcation, 1 = bifurcation)

$B_{6}$ elevation (m a.s.l)

$B_{7}$ stand layer to which the single tree belongs (0 = upper layer, 1 = understorey). All analysed trees belong to the upper layer.

The species-specific model parameters ($b_{i}$), as published in Herold *et al.*, (2019) are given for the Swiss Plateau in Appendix Table A2. The estimated stem volumes $\hat{Y}$ were then expanded to total tree AGB using the measured basic wood density and a tree type-specific (coniferous or deciduous) biomass expansion factor:


$$\begin{array}{l} AGB_{ALLOM_{NFI}}=\hat{Y}\times WD\times BEF \end{array}$$ (5)

where WD is the basic wood density and BEF is the biomass expansion factor (1.19 for coniferous and 1.31 for deciduous trees; Brändli, 2010) to expand stem volume over bark to total AGB.

## RESULTS

All 55 measured trees with information regarding their species, age, height, DBH and AGB (field-measured) are listed in Appendix Table A1. In the next sections, TLS-derived tree metrics are compared with reference values as listed in Table A1 based on traditional field inventory techniques. Additionally distribution of AGB within the tree and within the two compartments is analysed and the power of different TLS-derived tree metrics to estimate AGB is shown.

### DBH, tree height and crown variables

**Fig. 1.**
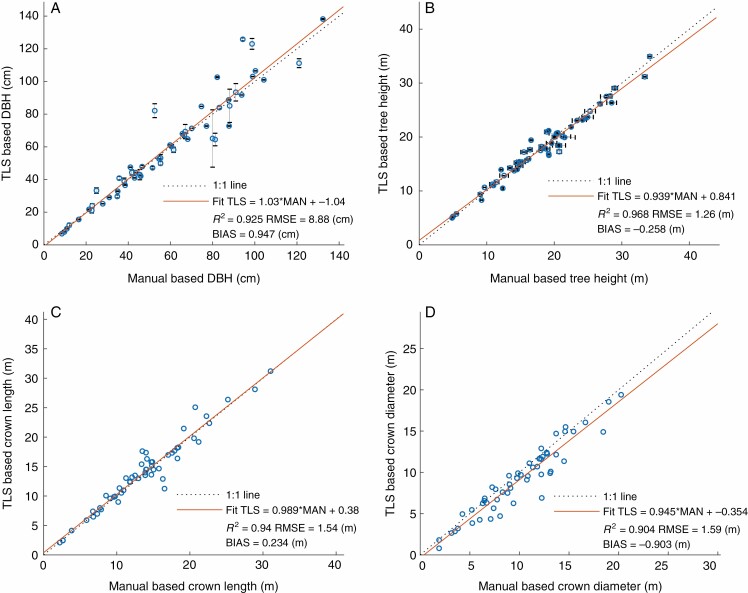
Traditional field measurement versus TLS-derived tree metrics: DBH (A), tree height (B), crown height (C) and crown diameter (D). TLS-based DBH (A) was derived from the cylinder fitted 1.3 m above ground; error bars denote the standard deviation due to the multiple QSM runs. Error bars in manually measured tree height (B) are due to multiple measurements of tree height (three measurements).

The TLS-derived DBH correlated well with the field measured DBH, resulting in an *R*^2^ of 0.925 and an RMSE of 8.88 cm (all RMSE values are with respect to the 1:1 line), as shown in Fig. 1A. The TLS-derived DBH was extracted from the diameter of the cylinder fitted to the point cloud 1.3 m above ground. The standard deviations of the TLS-derived DBH from the multiple QSM runs are shown as vertical error bars in Fig. 1A. Larger variations in DBH between QSM model runs indicate the sensitivity of the model output due to the extracted point cloud. Larger deviations between measured and TLS-derived DBH, however, can be caused by ivy (*Hedera helix*) growth on the tree trunk [overestimated DBH, e.g. Lausanne_15 with overestimation of 29.58 cm (56 %)] or due to trunk growth with multiple stems (e.g. Bern_16). The tree with the largest DBH variation for the multiple QSM runs [Bern_16, standard deviation of TLS-derived DBH: ±17.55 cm (26.9 %)] had four major trunks already separated <1.3 m above ground, making the identification of the main trunk as well as cylinder fitting difficult. This resulted in a mismatch between average TLS-derived DBH and reference DBH of 15 cm (18.5 %). Less circular tree trunks 1.3 m above ground (e.g. concave parts) can also cause deviations between TLS-derived and reference values and larger standard deviations in TLS-derived DBH.

Tree height derived directly from the TLS point cloud correlated well with the field-measured tree height, as shown in Fig. 1B, with an *R*^2^ of 0.968 and an RMSE of 1.26 m. The variation in manually measured tree height comes from multiple measurements and is therefore the standard deviation of the multiple tree height measurements.

Crown metrics derived directly from the TLS point cloud correlate well with field measurements, as shown in Fig. 1C, D for crown length (*R*^2^ = 0.94, RMSE = 1.54 m) and crown diameter (*R*^2^ = 0.904, RMSE = 1.59 m).

### Above-ground biomass

**Fig. 2.**
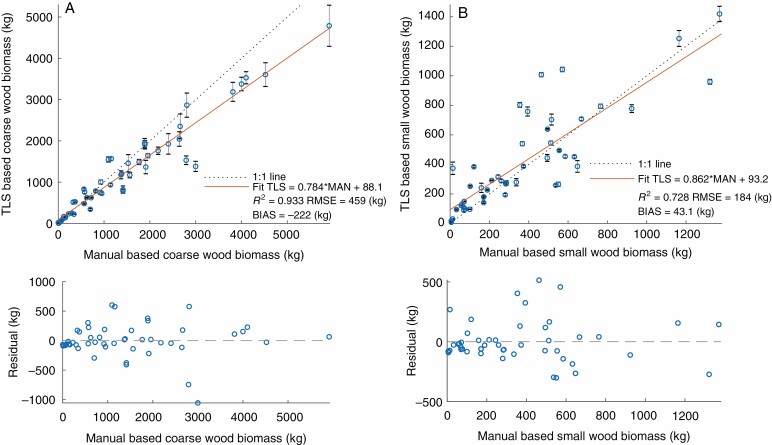
Manually measured coarse (A) and small (B) wood biomass versus TLS-derived biomass (threshold diameter >7 cm) (top row). Residuals of the fitted models (red line in top panels) are in the bottom row.

**Fig. 3.**
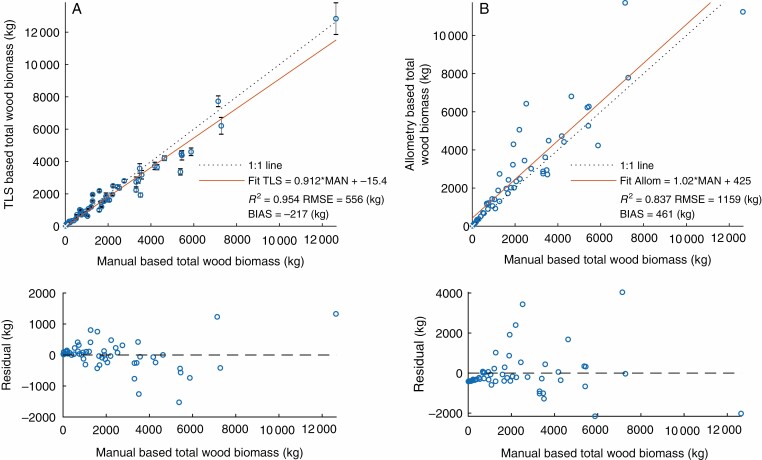
Manually measured total wood biomass versus total wood biomass derived by TLS (A) and allometry (B) (top row). Residuals of the fitted models (red line in top panels) are in the bottom row.

The TLS-derived AGB was compared with the destructively measured AGB for the coarse wood (diameter >7 cm; Fig. 2A) and small wood (diameter ≤7 cm; Fig. 2B) compartments, and for total wood AGB (Fig. 3A). The TLS-derived coarse wood AGB matches the destructively measured AGB well, with an *R*^2^ of 0.933 and an RMSE (with respect to the 1:1 line) of 459 kg. An underestimation of AGB for larger trees was observed, most probably caused by less circular trunks, where the QSM fits the cylinders within the trunk. The TLS-derived small wood biomass shows a weaker match with the destructively measured AGB, with an *R*^2^ of 0.728 and an RMSE with respect to the 1:1 line of 184 kg (Fig. 2B). Branch biomass seems to be slightly overestimated, especially for smaller and medium-sized trees. For total AGB, TLS is able to match the reference well, with an *R*^2^ of 0.954 and an RMSE (with respect to the 1:1 line) of 556 kg (Fig. 3A). The lower panels of Figs 2 and 3 show the residuals of the fitted model (red line in the upper panels), showing that the residuals are largely uncorrelated to tree size. Note that there are five additional trees (Winterthur_01, Winterthur_02, Zurich_01, Zurich_02, Zurich_03) included in the analysis of total AGB (Fig. 3A) than in the separate compartment analysis (Fig. 2), all of them showing on average larger AGB than the rest of the trees (Table A1). This is because the company cutting down the trees was not able to separate the two compartments due to time restrictions.

**Fig. 4.**
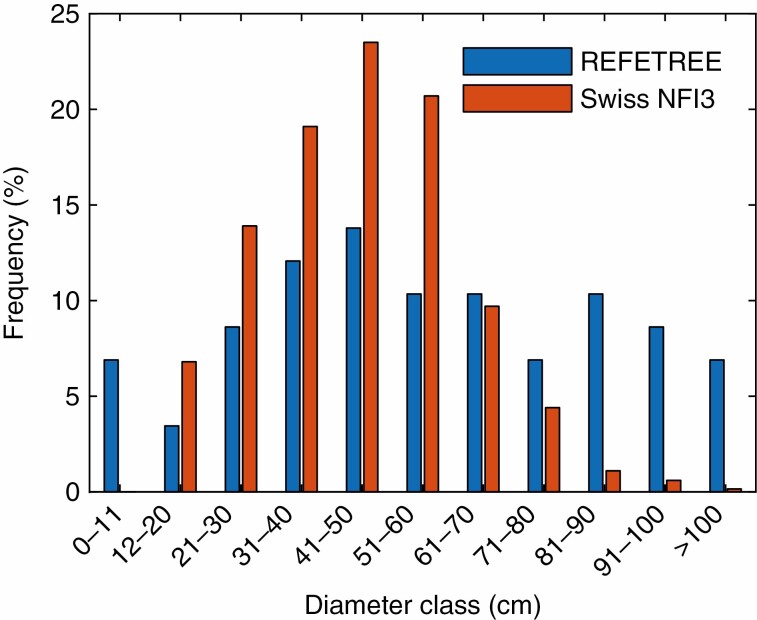
DBH frequency distribution for the urban trees measured in this study (REFETREE; blue, *n* = 55) compared with the DBH frequency distribution of the forest trees used to establish the allometric tariff models within the third iteration of the Swiss NFI (red, *n* = 16 628) (Abegg *et al.*, 2020b).

The TLS-derived AGB was compared with allometry-based estimates following species-specific allometric equations established by the Swiss NFI (Herold *et al.*, 2019). The fit between allometry-based AGB estimates and reference AGB [*R*^2^ = 0.837, RMSE = 1159 kg (with respect to 1:1 line)] is shown in Fig. 3B. Especially for medium to large trees, allometric AGB estimates based on tariff models of the Swiss NFI show larger deviations in estimated AGB, whereas TLS-derived AGB estimates show a closer match to the reference AGB measurements. Figure 4 shows the DBH frequency distribution of the urban trees measured in this study and the forest trees used to build up the allometric tariff models (data from Abegg *et al.*, 2020b), as described in *Comparison to Allometry based AGB estimates* in the Methods section. In this study the DBH distribution is shifted more to larger trees compared with the dataset in the Swiss NFI, potentially explaining part of the discrepancies in allometric AGB estimates for larger trees.

**Fig. 5.**
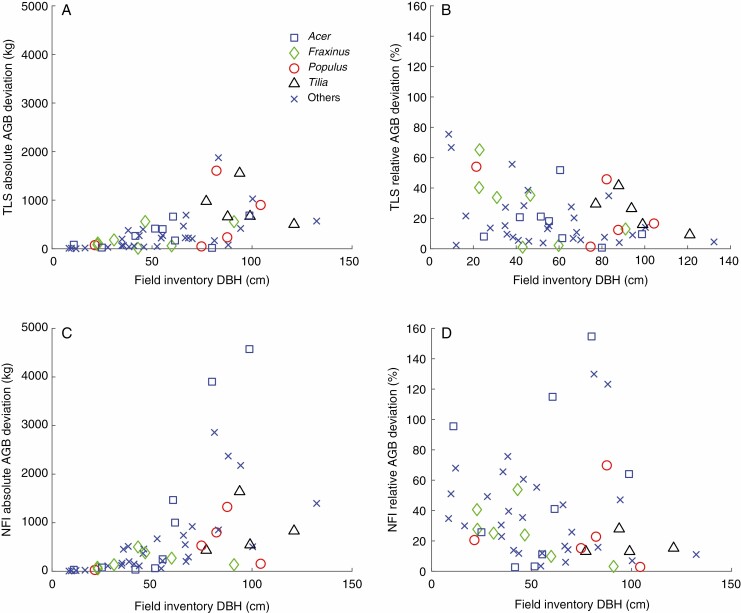
Absolute (A) and relative (B) tree AGB deviations for TLS-derived total wood AGB and for allometry-derived total wood AGB (absolute, C; relative, D). Allometries are based on the allometric tariff function of the Swiss NFI as published in Herold *et al.* (2019).

In Fig. 5 the absolute and relative AGB deviations from the reference measurements are given as a function of DBH for both TLS- and allometry-based AGB estimates. Tree genera with multiple samples in the acquired dataset are specifically highlighted. While TLS-derived AGB estimates show a clear pattern with respect to changing DBH (increasing absolute AGB deviation with increasing DBH, decreasing relative AGB deviation with increasing DBH), this pattern is less obvious in the allometry-derived estimates. Allometry-based AGB estimates also show larger absolute and relative deviations (average absolute and relative deviations, 674.7 kg and 33 %) throughout the whole DBH spectrum compared with TLS-derived AGB (average absolute and relative deviations, 350.4 kg and 24.9 %).

### AGB distribution within tree

**Fig. 6.**
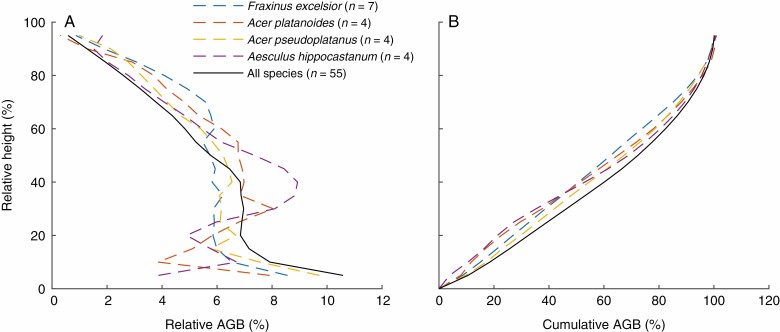
Relative and cumulative AGB distributions over normalized tree height.

**Fig. 7.**
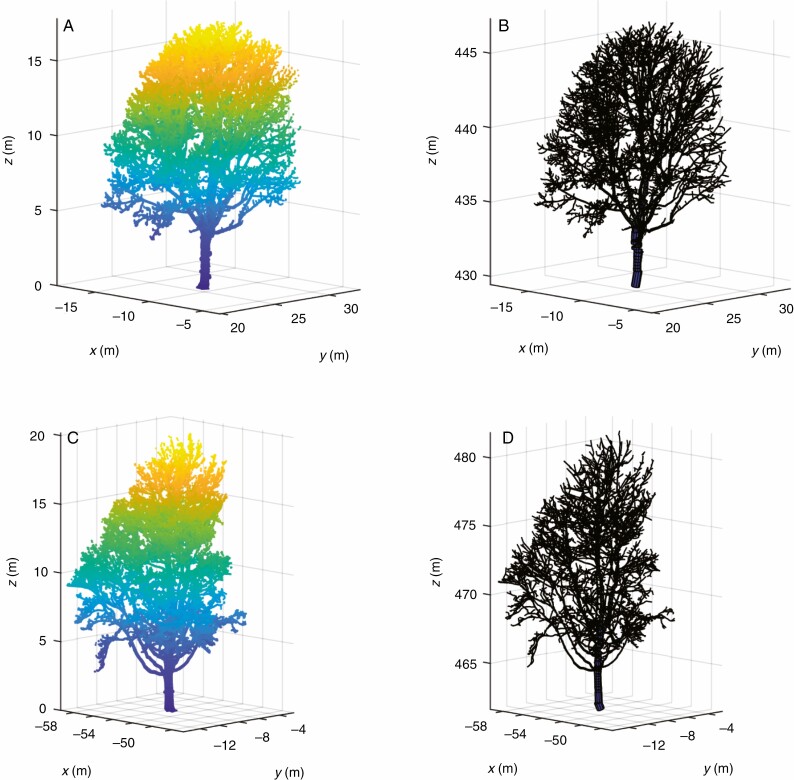
Point cloud (left) and QSM model visualization (right) for one individual of each of *Fraxinus excelsior* (Lausanne_03; A, B) and *Acer platanoides* (Lausanne_10; C, D), two of the four most common tree species in the acquired dataset.

**Fig. 8.**
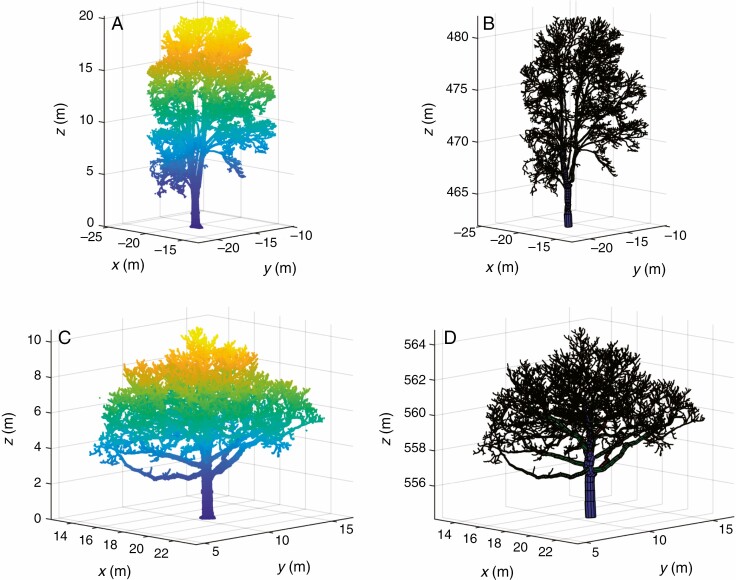
Point cloud (left) and QSM model (right) visualization for one individual of each of *Acer pseudoplatanus* (Lausanne_11; A, B) and *Aesculus hippocastanum* (Bern_01; C, D), two of the four most common tree species in the acquired dataset.

Terrestrial laser scanning provides the opportunity to analyse the AGB distribution as a function of tree height, as shown in Fig. 6. The distribution of AGB over tree height is shown for the four most common tree species in the dataset (*Fraxinus excelsior*, *Acer platanoides*, *Acer pseudoplatanus* and *Aesculus hippocastanum*), and the averaged height distribution for all species is shown in both relative and cumulative terms. Example point clouds and QSM models of these four tree species are shown in Figs 7 and 8. These results show that more AGB is stored in the lower parts of the trees. Averaged over all analysed individuals, 50 % of the AGB is approximately located in the lower 35 % of the tree and 80 % of the AGB is located in the lower 60 % of the tree. The AGB height distribution shows some clear discrimination between tree species. For example, *F. excelsior* trees show an increase in relative AGB above 60 % tree height (blue line in Fig. 6), a feature shown by no other analysed species. Such information could be exploited in, for example, automatic tree species classification from TLS data.

**Fig. 9.**
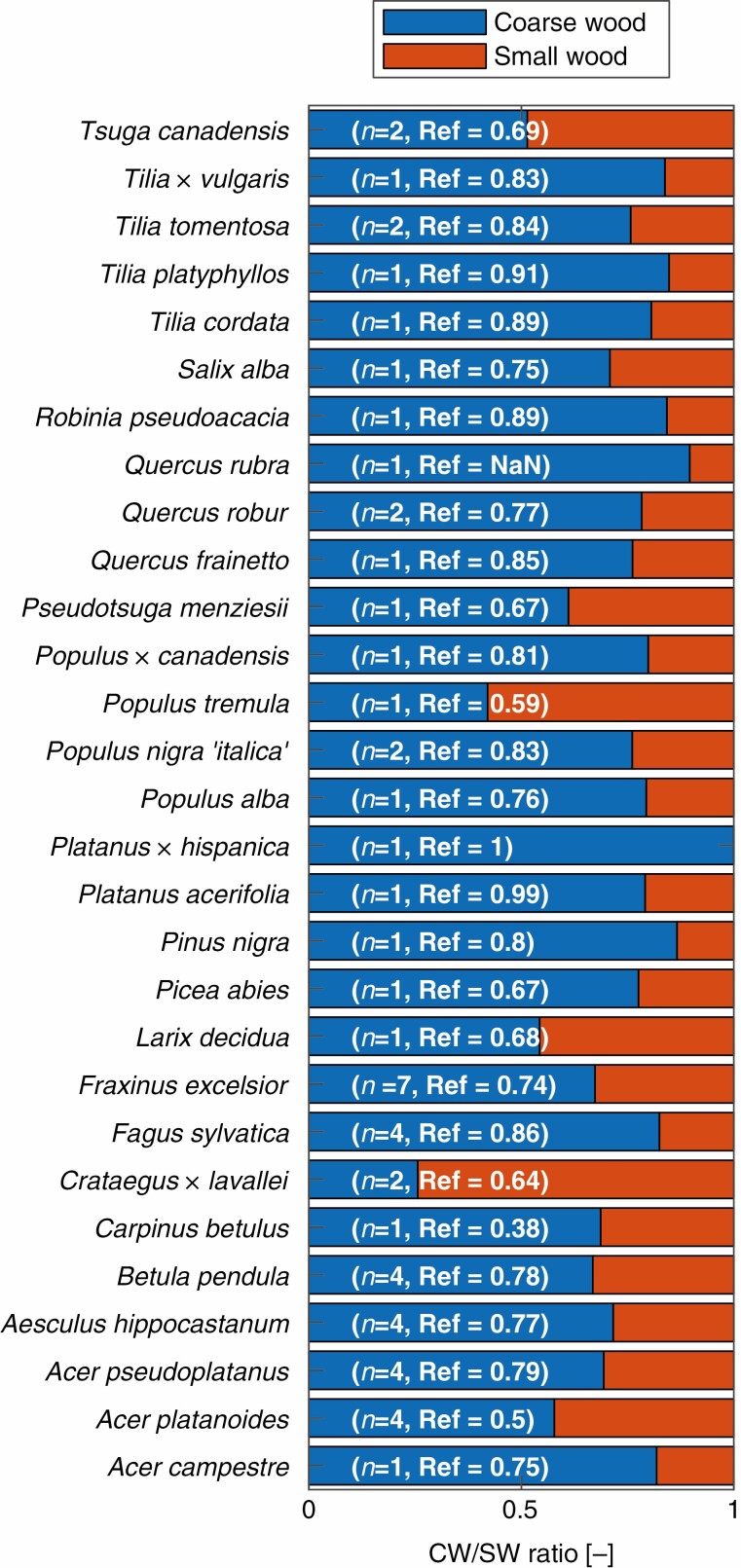
Coarse wood (diameter >7 cm) and small wood (diameter ≤7 cm) distribution aggregated by tree species. Number of trees per species and coarse wood/small wood (CW/SW) ratios of reference measurements are given in parentheses. If the reference value is missing (marked Ref = NaN in the figure) no separate weighting of the small wood and coarse wood compartment was performed. Trees with no coarse wood/small wood separation (Ref = NaN) are not included in the regression analysis for coarse and small wood (Fig. 2).

Additionally, TLS is able to accurately extract AGB for different compartments, as shown in Fig. 9, where the ratios of coarse wood to small wood aggregated by species are shown. The number of trees per species and the reference coarse wood/small wood (CW/SW) ratios are given in parentheses and show a good match to the TLS-derived CW/SW ratios (CW/SW ratio TLS, 72.5 %; reference, 76.5 %).

### AGB predictors

**Fig. 10.**
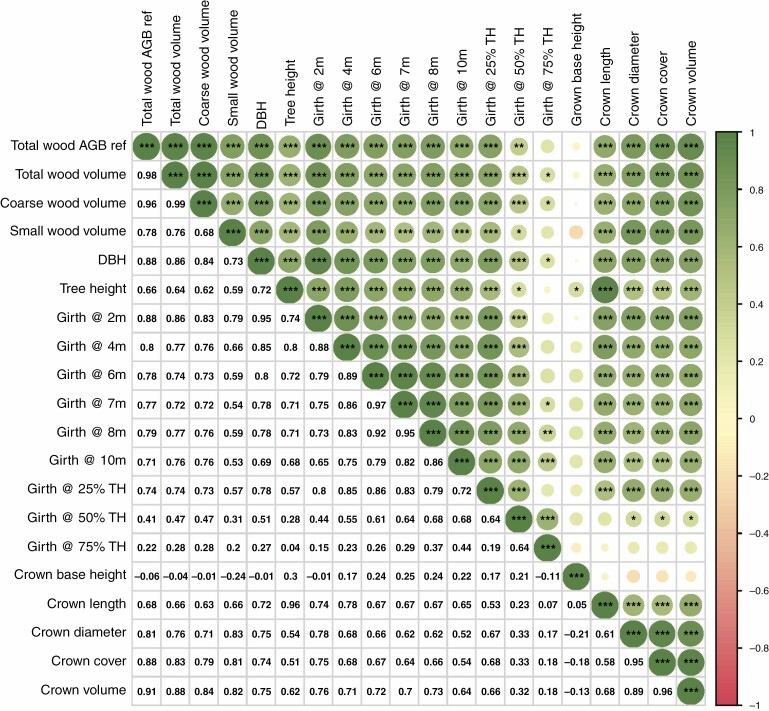
Cross-correlation of predictor variables with values at the lower left and and a symbolic representation of the cross-correlation on the upper right half of the table (circle size denotes degree of correlation; significance levels are ***0.001, **0.01, *0.05). TH, tree height.

As the acquisition and processing of high-resolution TLS point clouds to retrieve important tree metrics and AGB estimates is time-consuming and labour-intensive, it is of importance to quantify the predictive power of easily measurable tree metrics for estimating AGB. Due to the highly detailed tree reconstruction possible from TLS point clouds, we can extract, for example, stem diameters at multiple heights of the tree trunk and estimate the importance of these metrics in predicting AGB via a simple correlation analysis. We performed a correlation analysis using R’s corrplot package, in which we calculated the correlation coefficients relating reference total AGB to traditional field inventory metrics (i.e. DBH, tree height) and TLS-enabled tree metrics (i.e. wood volumes in different compartments, girth diameter at different heights of the trunk, crown dimensions). Figure 10 shows that total and coarse wood volume and crown volume have the highest correlations, of 0.98, 0.96 and 0.91, respectively (significance level 0.001); however, these are not easy to measure without a terrestrial laser scanner. These are followed by different trunk diameter measurements at lower heights above ground: DBH (0.88), girth at 2 m (0.88) and girth at 4 m (0.8) (significance level 0.001). Crown cover and diameter also show high correlation with AGB, with a correlation coefficient of 0.88 and 0.81, respectively. Tree height, a proxy relatively often used for AGB, shows a weaker correlation, of 0.66 (significance level 0.001). Crown base height and trunk diameters at or above 50 % tree height show the lowest correlation with AGB, of −0.06 and ≤0.41, respectively.

## DISCUSSION

In this study we reconstructed the 3-D structure of urban trees using TLS acquisitions at very high resolution and estimated AGB from the 3-D point cloud using a QSM modelling approach that was validated and compared with destructively acquired reference values and allometric estimates. In the following sections our findings and possible directions for further research are discussed.

### Tree metric extraction from TLS acquisitions

Terrestrial laser scanning has been established as a reference technique to extract very detailed and accurate structural information about complex forest canopies and single trees. It currently delivers 3-D point cloud data with the highest accuracy and level of detail at plot level for the extraction of forestry-relevant variables (Liang *et al.*, 2018; Piermattei *et al.*, 2019). Recent developments in the size and weight of these highly technological instruments, together with well advanced processing techniques, allow faster and more efficient acquisition and analysis of such 3-D data. Using the approach of QSM, accurate (within 10 % of values obtained for destructively harvested trees) wood volume and AGB estimates have been reported for a variety of tree species in both urban and forest environments (Lefsky and McHale, 2008; Calders *et al.*, 2015; Disney *et al.*, 2018; Gonzalez de Tanago *et al.*, 2018; Wilkes *et al.*, 2018). However, there are some limitations in the accurate retrieval of structural information on complex trees from TLS acquisitions. The positioning of the laser scanner relative to the objects can have a large impact on the completeness of the 3-D point cloud (Abegg *et al*., 2017), as occlusion due to obstructing branches or neighbouring objects can reduce the completeness of the retrieved tree model. Weather conditions can also affect the quality of the point cloud, especially reducing the fidelity of the point cloud for smaller- to medium-sized branches. Wilkes *et al.* (2018) found that even light winds lead to underestimation of wood volume, particularly towards the top of the canopy, where poorly resolved branches are not identified in the QSM. Nevertheless, we showed that TLS in combination with QSM is able to provide tree metrics (DBH, tree height, crown dimensions etc.) as well as AGB with good accuracy when compared with traditional forest inventory techniques. However, acquisition and processing times of TLS data still compare unfavourably with traditional techniques. However, with recent advances in hardware (i.e. smaller and lighter TLS instruments such as the Leica BLK360, Leica Geosystems, Heerbrugg, Switzerland) and software development to automate many processing steps (i.e. automatic co-registration of point clouds of different scans), these limitations are likely to become ever smaller in the near future. Also, the application of machine and deep learning methods to TLS point clouds could bring new methods for faster and more automatic retrieval of tree structural information (e.g. Wang *et al.*, 2017; Anderson *et al.*, 2018; Rehush *et al.*, 2018) and could show potential in overcoming current limitations of TLS applications. Additionally, with the acquisition of TLS point clouds it is possible to extract many additional relevant tree metrics (e.g. trunk diameters at different heights) that are either impossible to obtain by traditional inventory techniques or would be too labour-intensive. Furthermore, these point clouds can be stored and evaluated again with new and improved processing techniques in the future.

Larger deviations in DBH and AGB can be caused by ivy coverage on the tree trunk, obstructing a clear view of the trunk, possibly resulting in overestimation of both DBH and AGB for the affected trees. Even though most TLS acquisitions were performed during leaf-off conditions, a few trees still had significant foliage on them, resulting in occluded areas of the crown, making the accurate modelling of the branch structure and upper parts of the trunk difficult. Recent studies have shown that leaves and needles could be extracted from the point cloud based on a geometrical and/or radiometric filtering procedure (Moorthy *et al.*, 2019; Vicari *et al.*, 2019; Vicari, 2019; Wang *et al.*, 2020). However, these methods can fail if the foliage cover is too dense and the TLS device is thus not able to penetrate the outer foliage envelope, resulting in incomplete coverage of the inner branching structure. We therefore suggest TLS acquisition for accurate AGB estimates should be restricted to leaf-off conditions. Similar problems can be found for coniferous trees, where needles obstruct the view of the branching structure. However, due to the often relatively regular growing patterns of coniferous trees, with a strong and linear growing trunk and rather small branches, simple allometric equations can already result in accurate AGB estimates.

### Reference acquisition

Acquisition of field references (tree height, DBH, crown dimensions, AGB etc.) has been performed by varying field teams and contractors, and thus a potential bias caused by different personnel or weighing equipment cannot be eliminated. Furthermore, for a few trees no separation of small and coarse wood was performed due to time restrictions.

The determined basic wood density, which can affect estimated AGB from TLS-derived wood volume, corresponds well with values found in the literature (Chave *et al.*, 2009). However, our analysis showed high intraspecific variation (Table 1), *F. excelsior* being the species with the highest variation in basic wood density, ranging from 0.52 to 0.69 g cm^−3^.

**Table 1.** Measured wood densities compared with literature values. Values in brackets denote observed minimum and maximum values of the samples or values in published literature

Species	Samples	Wood densities of samples (g cm^−3^)	Wood densities in literature (g cm^−3^)
*Acer campestre*	1	0.56	0.53
*Acer platanoides*	4	0.57 (0.54–0.62)	0.52 (0.51–0.53)
*Acer pseudoplatanus*	4	0.56 (0.49–0.59)	0.51
*Aesculus hippocastanum*	4	0.46 (0.42–0.49)	0.50
*Betula pendula*	4	0.52 (0.46–0.57)	0.51 (0.50–0.53)
*Carpinus betulus*	2	0.62 (0.61–0.63)	0.69 (0.68–0.71)
*Crataegus* sp.	2	0.57 (0.56–0.58)	0.66 (0.62–0.70)
*Fagus sylvatica*	4	0.59 (0.53–0.68)	0.59
*Fraxinus excelsior*	8	0.58 (0.52–0.69)	0.57 (0.56–0.59)
*Larix decidua*	1	0.52	0.47
*Picea abies*	1	0.38	0.37
*Pinus nigra*	2	0.51 (0.49–0.53)	0.42 (0.41–0.42)
*Patanus* sp.	3	0.48 (0.47–0.48)	0.50 (0.37–0.60)
*Pupulus nigra*	2	0.35 (0.34–0.36)	0.35
*Populus* sp.	3	0.43 (0.41–0.44)	0.37 (0.35–0.39)
*Pseudotsuga menziesii*	1	0.58	0.43 (0.40–0.45)
*Quercus frainetto*	1	0.68	–
*Quercus robur*	2	0.60 (0.55–0.64)	0.58 (0.52–0.62)
*Quercus rubra*	1	0.50	0.56
*Robinia pseudoacacia*	2	0.63 (0.61–0.65)	0.68 (0.66–0.70)
*Salix alba*	1	0.39	0.28
*Tilia* sp.	6	0.43 (0.38–0.45)	0.42
*Tsuga canadensis*	2	0.42 (0.38–0.45)	0.38
*Ulmus minor*	1	0.52	0.55

### TLS versus allometry

We show that TLS-derived AGB estimates match the destructively harvested reference values better (*R*^2^ of 0.954 versus 0.837 and RMSE of 556 versus 1159 kg) than estimates based on species-specific empirical allometric equations established in the framework of the Swiss NFI (Herold *et al.*, 2019). However, these allometric equations were established for forest trees, which can show significant differences in form and dimension compared with their non-forest counterparts (McHale *et al.*, 2009). As shown in Fig. 4, the DBH distribution for the urban trees analysed in this study is shifted towards larger trees compared with the DBH distribution found in the forest trees used to establish the allometric tariff models within the Swiss NFI (Herold *et al.*, 2019). This can partially explain the discrepancies with respect to the reference values for the allometrically derived AGB estimates for the larger trees. Another contributor to the discrepancies might be differing growth conditions and competition. Gardi *et al.* (2016) showed that forest trees grow higher for a given DBH compared with isolated urban trees, also resulting in increased AGB, possibly explaining the overestimation of allometric AGB estimates for larger urban trees. It is therefore important to establish new allometric equations specifically for urban trees. The accuracy achievable with TLS in deriving tree structure and AGB information independent of tree size and form could lead to a new approach to the establishment of new allometric equations without the need for destructive sampling. Especially for urban trees, for which allometric equations are still rare, the establishment of new models based on TLS acquisitions could be promising. Roxburgh *et al.* (2015) reported that at least 50 samples per tree species would be needed to establish a robust allometric equation. The TLS-based establishment of new allometric equations could therefore reduce the number of harvested trees substantially, to only a few for calibration and validation of TLS-derived models.

Because many additional tree variables beyond the typically measured DBH and tree height can be derived from TLS acquisitions, it was possible to test which explanatory variables are best to predict AGB by using a simple correlation analysis (see AGB predictors in the Results section). This analysis showed that, apart from total and coarse wood volumes, trunk diameters at the lower part of the trunk show the highest predictive power (diameter at 2 m above ground and DBH), as well as crown dimension metrics. Interestingly, though, tree height has rather low predictive power compared with different trunk diameters and crown metrics. Due to different environmental conditions (different stress, lower direct competition with neighbouring trees, different management practices etc.), urban trees might show larger variation in height within the same tree species, possibly making tree height a less powerful predictor of AGB in an urban environment. This analysis further shows that the outline of the lower trunk (i.e. the trunk curve) could be a suitable predictor of AGB. This could make terrestrial photogrammetry with structure from motion (SfM) approaches a suitable and cost- and time-effective alternative to the more time-consuming TLS or traditional field inventory acquisitions (Iglhaut *et al.*, 2019; Piermattei *et al.*, 2019).

### Conclusions and outlook

Monitoring urban trees is becoming increasingly important, especially with the increase in urban area in mind. Non-destructive assessment of the structure and AGB of urban trees is therefore of increasing importance in quantifying the value of the large variety of ecosystem services they can provide. As empirical allometric equations developed for forest environments may not be transferable to urban trees, it is of great importance to develop accurate and robust tools to measure and monitor urban AGB and other tree structure metrics. Remote sensing and LiDAR technologies in particular have shown promising potential to measure and assess 3-D tree structure and AGB at unprecedented levels of detail. Due to the large variability in urban tree structure caused by highly diverse environmental conditions, accurate and highly detailed assessment of tree structure is needed. Terrestrial laser scanning has shown large potential for the assessment of urban tree structure, AGB and hence carbon sink. For this reason TLS has been added to the 2019 refinement of the 2006 IPCC Guidelines for National Greenhouse Gas Inventories as a promising new technology (Ogle *et al.*, 2019). In this study we assess the capacity and accuracy of TLS-based methods to extract AGB and tree structure metrics independent of tree species, size and form. Even though TLS acquisitions with the approach presented here are still time-consuming, extensive and highly detailed TLS scans of a large variety of trees could help in developing better allometric equations to more accurately estimate urban AGB based on a few easily measurable tree metrics (e.g. DBH, trunk diameters at different heights and crown dimensions). Advances in laser scanning technologies (i.e. development of mobile as well as drone-based laser scanners) and the increasing automation of point-cloud processing (i.e. automatic coregistration of point clouds) could further help with large-scale urban AGB monitoring. Overall, advances in remote sensing have introduced new ways of assessing forest and tree structural metrics even beyond AGB, giving us new and interesting insights into tree structure, architecture and functioning.

## APPENDIX

### A.1 LIST OF MEASURED TREES

In Table A1 the list of all measured trees with summary statistics of TLS and reference values for tree height, DBH, coarse wood, small wood and total AGB is shown.

A.2 TARIFF MODELS FOR STEM VOLUME OF THE SWISS NFI

**Table A1.** List of measured trees. Trees with unknown age are marked NA. TLS-derived and manually measured reference values are given for tree height, DBH, coarse (AGB_CW_) and small wood (AGB_SW_) compartments (threshold at 7 cm diameter) and total AGB (AGB_tot_)

Tree ID	Species	Age (years)	Height TLS/Ref (m)	DBH TLS/ Ref (cm)	AGB_CW_ TLS/ Ref (kg)	AGB_SW_ TLS/ Ref (kg)	AGB_tot_ TLS/ Ref (kg)
Bern_01	*Aesculus hippocastanum*	83	10.7/9.7	52.7/54.6	1182/1383	264/283	1447/1667
Bern_02	*Tilia* × *vulgaris*	NA	21.9/22.5	111.2/121	4143/4522	801/924	4944/5446
Bern_04	*Fraxinus excelsior*	60	19.4/16.6	29/31	536/369	186/171	722/540
Bern_05	*Fraxinus excelsior*	40	17.2/15.5	41/43	629/718	291/212	920/930
Bern_06	*Tilia platyphyllos*	125	20.8/20.5	103/99	2977/3812	535/368	3512/4179
Bern_07	*Crataegus* × *lavallei*	18	5.3/5	7.9/9.7	9/13	21/6	30/18
Bern_08	*Crataegus* × *lavallei*	15	5/4.9	6.8/8.5	5/8	19/6	24/14
Bern_09	*Acer pseudoplatanus*	120	15.4/15.2	60.2/60.5	1540/1156	398/121	1938/1277
Bern_10	*Fraxinus excelsior*	73	11.2/10.8	23.9/22.9	171/120	128/60	299/180
Bern_11	*Fraxinus excelsior*	185	21.2/19.2	93.4/91	3453/3999	275/286	3727/4285
Bern_12	*Betula pendula*	67	17.5/17.9	47.5/41	736/818	282/259	10 184/1078
Bern_14	*Aesculus hippocastanum*	24	5.2/4.97	12.1/12	14/16	8/6	22/23
Bern_15	*Platanus* × *hispanica*	75	13.9/12.1	44.2/43.6	679/949	0	679/949
Bern_16	*Acer campestre*	50	15.7/15.9	65.1/80	2050/1890	455/632	2505/2523
Bern_17	*Larix decidua*	40	17.6/16.4	42.9/45.5	346/700	291/337	637/1037
Biel_01	*Populus nigra ‘italica’*	65	34.9/34.1	102.6/82.2	1349/2999	554/512	1904/3511
Biel_02	*Pseudotsuga menziesii*	91	29.1/29	71.3/70.1	2053/2403	1308/1164	3361/3568
Biel_03	*Betula pendula*	NA	18.9/19.6	49.9/55.3	754/1412	779/394	1533/1806
Biel_04	*Fagus sylvatica*	83	26.2/26.8	106.5/100.3	4819/5913	1437/1370	6257/7283
Biel_05	*Fagus sylvatica*	70	23.7/24.9	40.8/35.7	649.7/622.6	108/69	758/692
Biel_06_01	*Tilia tomentosa*	100	18.2/18.1	72.7/77.1	1644/2791	688/516	2332/3307
Biel_06_02	*Tsuga canadensis*	93	14.7/14.8	25.2/27.9	142/156	115/70.03	257/226
Biel_06_03	*Tsuga Canadensis*	90	15.9/16.2	36.5/38.5	221/343	243/159	463/502
Biel_07	*Picea abies*	93	27.5/27.7	68/65.7	950/1140	275/551	1225/1691
Lausanne_01	*Fraxinus excelsior*	24	11/11	21/22.7	134/133	153/71	287/205
Lausanne_02	*Fraxinus excelsior*	NA	18.4/20.7	61.1/59.8	1664/2179	1027/571	2691/2750
Lausanne_03	*Fraxinus excelsior*	57	17.8/18.8	47.9/46.6	1514/1095	636/495	2150/1591
Lausanne_04	*Populus alba*	NA	26.4/28.5	101/104.3	3588/4098	931/1321.69	4519/5420
Lausanne_05	*Quercus robur*	70	17.9/18.3	69.3/67	1934/2644	782/766	2717/3409
Lausanne_06	*Acer pseudoplatanus*	28	16.9/19.2	33.2/24.9	237/226	90/77	327/303
Lausanne_07	*Quercus robur*	47	19.9/21.5	32.8/35	796/560	135/171	931/731
Lausanne_08	*Populus* × *Canadensis*	NA	27.6/28.3	84.7/74.7	2814/2807	709/668	3523/3475
Lausanne_09	*Populus nigra ‘italica’*	35	23.1/23.4	88.7/87.6	1737/1526	398/373	2135/1900
Lausanne_10	*Acer platanoides*	41	20/20	47.2/51.5	1073/1370	467/585	1540/1955
Lausanne_11	*Acer pseudoplatanus*	NA	20.1/21.2	58.2/61.5	1774/1886	496/555	2271/2441
Lausanne_12	*Robinia pseudoacacia*	NA	16.6/19.2	64.5/81.2	1713/1957	320/244	2034/2201
Lausanne_14	*Platanus acerifolia*	NA	8.3/9.2	85.1/88.1	1461/1911	385/14	1847/1925
Lausanne_15	*Betula pendula*	41	17.3/20.7	82.1/52.55	960/929	203/280	1163/1209
Lausanne_16	*Carpinus betulus*	NA	10.5/12.4	multiple trunks	592/325	270/537	862/863
Luzern_05	*Betula pendula*	NA	14/14.5	15.6/16.5	55/59	32/13	88/72
Luzern_09	*Acer pseudoplatanus*	NA	15.4/14.8	44.3/41.7	731/917	804/354	1535/1270
Luzern_10	*Aesculus hippocastanum*	100	12.9/12.6	64.7/68.2	1351/1562	483/494	1834/2056
Luzern_13	*Acer platanoides*	NA	13.8/14.1	53.2/55.3	1621/1765	1013/464	2634/2228
Luzern_14	*Acer platanoides*	NA	5.7/5.5	9.9/10.9	20/0	94/32.38	115/32
Luzern_15	*Quercus frainetto*	35	15.2/14.4	38.9/38.1	803/575	251/102	1054/678
Solothurn_01	*Salix alba*	NA	14.3/13.4	29.6/34.8	238/297	98/100	336/397
Solothurn_02	*Populus tremula*	20	11.7/11.5	21.7/21.4	87/80	122/57	211/137
Solothurn_04	*Aesculus hippocastanum*	40	9.4/9	42/45.9	488/567	231/189	719/756
Solothurn_05	*Tilia cordata*	75	20.9/18.9	72.7/87.8	746/1413	180/171	926/1584
Solothurn_06	*Pinus nigra*	NA	27.8/25.3	67.2/66.6	2672/2660	412/648	3084/3308
Winterthur_01	*Tilia tomentosa*	86	23.2/24.2	91.7/93.8	3498/-	821/-	4319/5874
Winterthur_02	*Quercus rubra*	149	31.2/33.4	138.2/132.3	1184/–	1366/–	13 209/12 640
Zurich_01	*Fagus sylvatica*	78	20.7/20.3	125.8/94.3	3812/–	399/–	4211/4629
Zurich_02	*Fagus sylvatica*	68	18.5/20.7	83.9/83.2	2687/–	814/–	3501/5376
Zurich_03	*Acer platanoides*	68	23.5/24.5	123/98.7	6394/–	1432/–	7826/7140

In Table A2 the coefficients for the species specific tariff models for stem volume (see Equation (4)) as used in the Swiss NFI are shown. Only the parameters of the tariff functions of the species occurring in this study are shown. For a full list, please refer to (Herold et al., 2019).

**Table A2.** Coefficients of the tariff models for stem volume used in the Swiss NFI (taken from Herold *et al.*, 2019). Variables marked – ^a^ are not part of the allometric model

Species	b0	b1	b2	b3	b4	b5	b6	b7
Norway spruce (*Picea abies*)	−11.433229	3.5068775	−0.0061792	1.0366E−05	0.0052032	−0.2891386	−0.0001455	− ^a^
Silver fir (*Abies alba*)	−9.1920196	2.7776135	−0.0033026	1.1998E−05	0.0032396	−0.0954103	−9.42E−05	−0.0799185
Pine (*Pinus sylvestris*, *P. nigra*, *P. mugo arborea*)	−10.94328	3.2717589	−0.0047869	2.6183E−05	0.0020279	− ^a^	− ^a^	− ^a^
Larch (*Larix decidua*)	−11.820115	3.6012538	−0.0064718	3.8231E−05	0.003826	−0.1613262	0.0001787	− ^a^
Other conifers	−11.669887	3.6109797	−0.0060115	− ^a^	0.0052156	−0.1916867	−0.000475	− ^a^
European beech (*Fagus sylvatica*)	−11.08317	3.3529562	−0.0048976	2.3704E−05	0.0011135	−0.1103548	− ^a^	−0.3674877
Oak (all species) (*Quercus* spp.)	−11.240067	3.3752972	−0.0053652	− ^a^	0.004923	−0.1366446	− ^a^	− ^a^
Sycamore and Norway maple (*Acer pseudoplatanus*, *A. platanoides*)	−7.8080883	2.1328846	− ^a^	4.0574E−05	0.0067504	−0.1411788	−0.000194	−0.2800938
Ash (*Fraxinus excelsior*, *F. ornus*)	−14.796222	4.7069939	−0.0110384	− ^a^	− ^a^	−0.1001387	− ^a^	− ^a^
Chestnut (*Castanea sativa*)	−6.4107181	1.7651895	− ^a^	− ^a^	− ^a^	− ^a^	−0.0004751	−0.2959489
Other broadleaved	−9.9483742	2.9543902	−0.00362	− ^a^	0.0012139	−0.1706129	− ^a^	−0.1849696
